# The second complete mitochondrial genome of *Capillidium rhysosporum* within the family Capillidiaceae, Entomophthorales

**DOI:** 10.1080/23802359.2024.2324938

**Published:** 2024-03-11

**Authors:** Hanwen Lu, Yong Nie, Bo Huang

**Affiliations:** a Anhui Provincial Key Laboratory for Microbial Pest Control, Anhui Agricultural University, Hefei, China; b School of Civil Engineering and Architecture, Anhui University of Technology, Ma’anshan, China

**Keywords:** *Capillidium*, Entomophthromycotina, mitochondrion, phylogenetic analyses

## Abstract

The complete mitochondrial genome of the entomophthoroid fungus *Capillidium rhysosporum* (strain no.: ATCC 12588) was sequenced using next-generation sequencing technology. The assembled circular genome has a length of 46,756 base pairs with a GC content of 27.06%. Gene prediction identified 15 core protein-coding genes (PCGs), two rRNA genes, and 27 tRNA genes. Phylogenetic analysis confirmed that *C. rhysosporum* belongs to the Zoopagomycota clade and is closely related to *C. heterosporum*. This study presents the second complete mitochondrial genome within the family Capillidiaceae, contributing to the mitochondrial DNA database of entomophthoroid fungi.

## Introduction

The genus *Capillidium* B. Huang & Y. Nie 2020 was recently distinguished from *Conidiobolus* (Brefeld 1884) based on its unique characteristic of capilliconidia arising from conidia (Nie et al. 2020). Subsequently, it was reclassified into the family Capillidiaceae Y. Nie, Stajich & K.T. Hodge 2022 based on molecular data, ancestral lifestyles, and morphological features (Gryganskyi et al. 2022). With the addition of two more species in 2022, there are now 10 known species in this genus (Nie et al. 2022).

In a previous study, we reported the mitochondrial genome of *Capillidium heterosporum* (Drechsler) B. Huang & Y. Nie 2020 as the first complete mitochondrial genome in the family of Capillidiaceae. Comparative analysis revealed it to have the second-highest number of introns among 22 basal fungi (Nie et al. 2019). Here, we present the sequencing and analysis of the second complete mitochondrial genome of Capillidiaceae, providing insights into phylogenetic relationships and evolutionary histories among entomophthoroid fungi.

## Materials and methods

**Figure 1.**
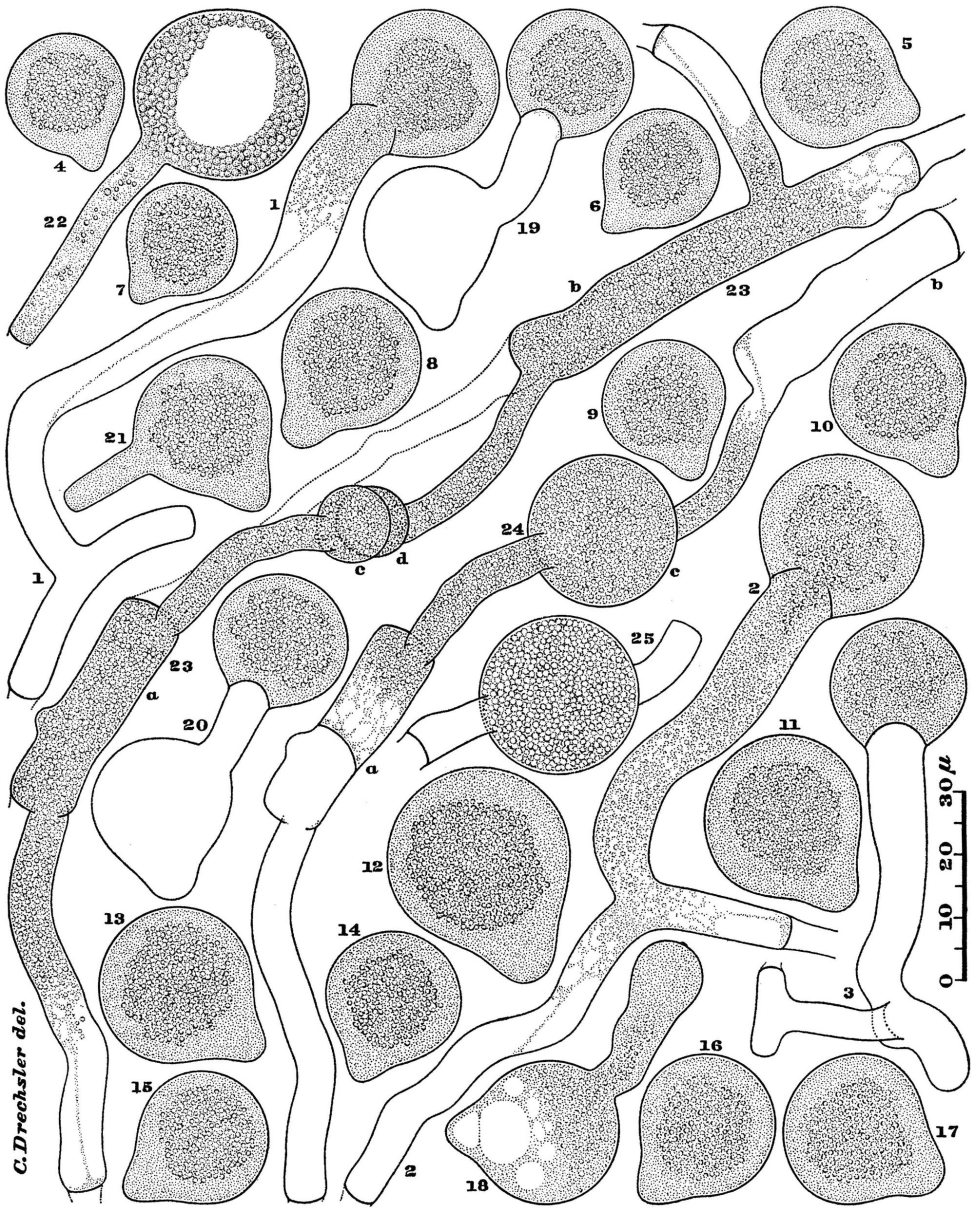
*Capillidium rhysosporum* as found in maize-meal agar cultures; ×1000. (1–3) Stout conidiophores bearing globose conidia. (4–17) Detached globose conidia. (18–20) Conidia in two stages of repetitional development. (21, 22) Conidia germinating on agar already permeated with mycelium of fungus. (23) Hypha with two non-adjacent segments, a and b, that are ready to conjugate by union of their terminally swollen branches, c and d. (24) Same, 45 min. Later, the two segments, a and b, now nearly empty from migration of contents into the enlargement c. (25) Same, after another interval of 45 min; young zygospore now full-grown (Drechsler 1954).

The ex-type strain ATCC 12588 of *C. rhysosporum* was obtained from the American Type Culture Collection (Manassas, VA), and this species was known for its rough zygospores, and was isolated from decaying plant debris near Laplace (38°32′ N, 76°59′ W), Louisiana, United States in 1952 (the original descriptive image were shown in Figure 1). Fungal cultures were incubated on PDA for seven days at 21 °C. Total genomic DNA was extracted from fresh fungal mycelia using a modified CTAB method (Watanabe et al. 2010). Duplicate specimens and genomic DNA were deposited at the Research Center for Entomogenous Fungi (RCEF), Anhui Agricultural University (www.ahau.edu.cn, Professor Bo Huang, Email: bhuang@ahau.edu.cn), Hefei, Anhui, China under the voucher number RCEF 20081012. The constructed paired-end libraries with 300 bp inserts was used for Illumina HiSeq X-ten sequencing (NextOmics Biosciences, Co., Ltd., Wuhan, China) following the manufacturer’s instructions (Bioo Scientific, Austin, TX, AIR™ Paired-End DNA Sequencing Kit). Quality assessment was performed to obtain clean reads from the raw sequencing data by FastQC 0.11.8 (Andrews 2010). The sequencing depth and coverage map were provided in the supplementary materials (Supplementary Figure S1). The mitogenome was assembled using Norgal 1.0 (Al-Nakeeb et al. 2017), and gene annotation was conducted as previously described (Zhang et al. 2017; Li et al. 2021), with tRNA genes identified using tRNAscan-SE v1.3.1 (Lowe and Eddy 1997). Circular mitogenome maps were drawn with PMGmap (Zhang et al. 2023, http://www. 1kmpg.cn/pmgmap). CPGView (http://www.1kmpg.cn/cpgview/) was utilized to illustrate the structures of intron-containing genes as shown in Supplementary Figure S2.

Mitochondrial genome sequences from fungi in four phyla of Blastocladiomycota, Chytridiomycota, Mucoromycota, and Zoopagomycota were downloaded from GenBank, with two Ascomycetes (*Yarrowia lipolytica* and *Colletotrichum acutatum*), and one Basidiomycete (*Lactifluus hygrophoroides*) were chosen as outgroups. A combined mitochondrial gene dataset of 14 proteins (*atp6*, *atp8*, *atp9*, *nad1*, *nad2*, *nad3*, *nad4*, *nad4L*, *nad5*, *nad6, cob, cox1*, *cox2*, and *cox3*) was used for phylogenetic analysis. Individual amino acid sequences were aligned using MEGA v6.06 (Caspermeyer 2016) and then concatenated with SequenceMatrix v1.7.8 (Vaidya et al. 2011). The best model, GTR + G + I, for the maximum-likelihood (ML) analysis was selected based on the Akaike information criterion (AIC) using Modeltest 3.7 (Posada and Crandall 1998). A phylogenetic tree using the ML method was constructed with RAxML 8.1.17 with 1000 bootstrap replicates (Stamatakis 2014). The BI analysis was performed with MrBayes 3.2.2 (Ronquist and Huelsenbeck 2003). Phylogenetic trees were modified with FigTree 1.4 (Rambaut 2012).

## Results

**Figure 2.**
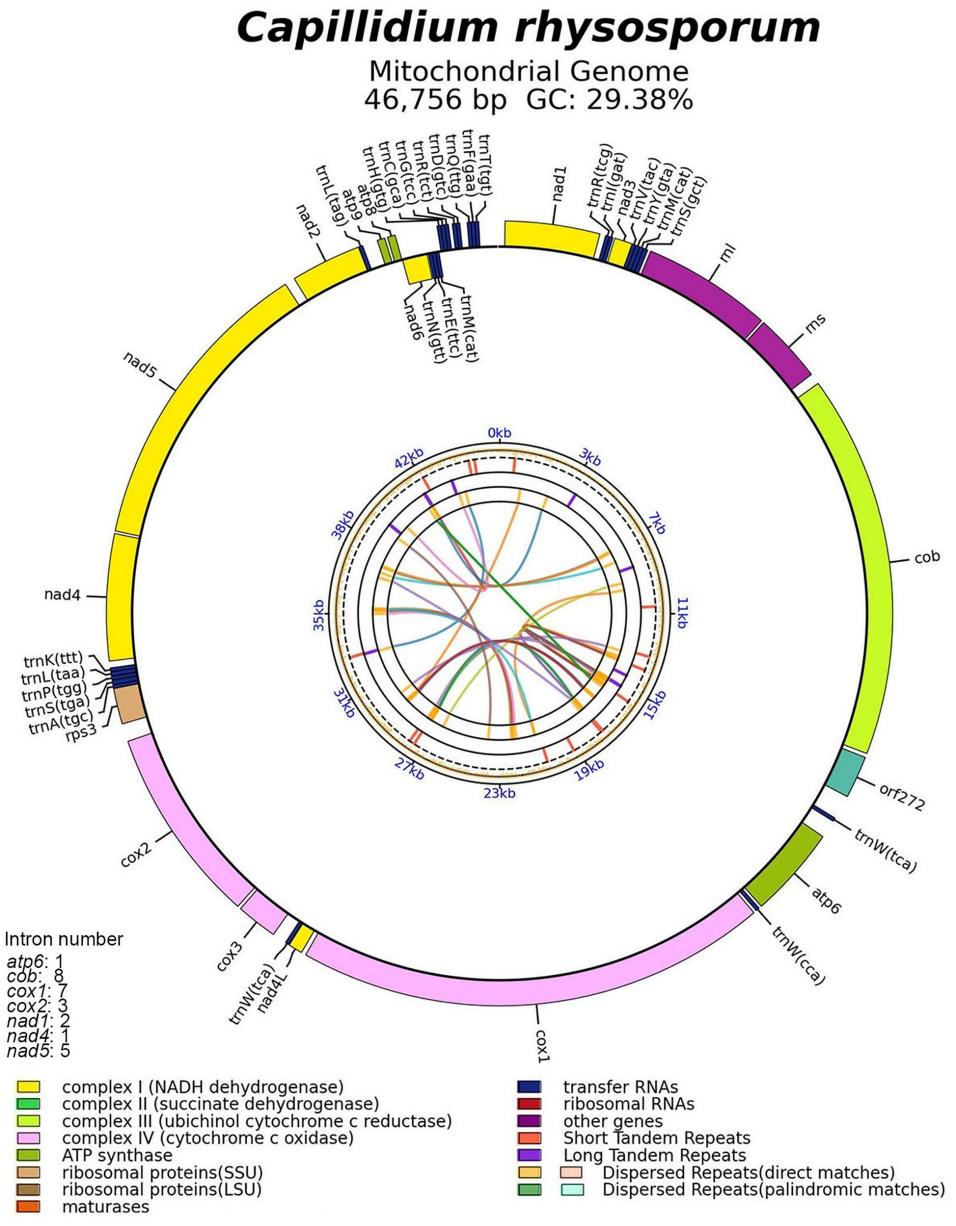
Circular complete mitogenome map of *Capillidium rhysosporum* genes is represented by different colored blocks. Genes outside and inside the outermost black ring line are transcribed in a clockwise and counter-clockwise direction, respectively. The inner circles represent the genome scale, GC content and distributions of short tandem repeats, long tandem repeats and the dispersed repeats, respectively. The colored parabola in the center circle represents the dispersed repeats.

**Figure 3.**
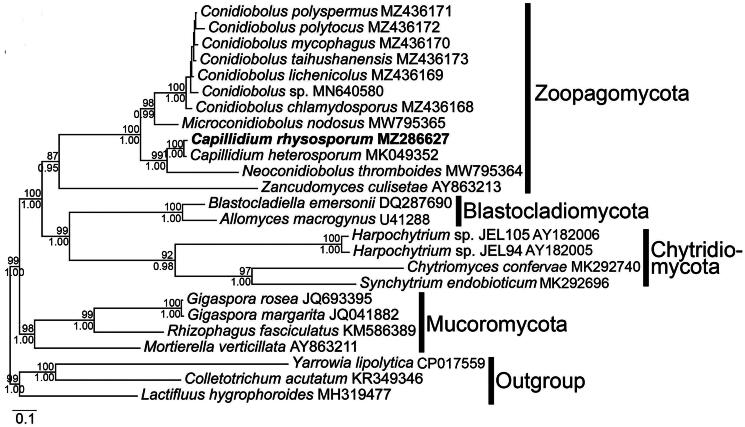
The phylogenetic tree based on 14 mitochondrion-encoded proteins. The 25 fungal mitogenomes were used in this phylogenetic analysis: *Allomyces macrogynus* (U41288, Paquin and Lang 1996), *Blastocladiella emersonii* (DQ287690, Tambor et al. 2008), *Capillidium rhysosporum* (MZ286627, this article), *Capillidium heterosporum* (MK049352, Nie et al. 2019), *Chytriomyces confervae* (MK292704, van de Vossenberg et al. 2018), *Conidiobolus chlamydosporus* (MZ436168, Nie et al. 2021), *Conidiobolus lichenicolus* (MZ436169, Nie et al. 2021), *Conidiobolus mycophagus* (MZ436170, Nie et al. 2021), *Conidiobolus polyspermus* (MZ436171, Nie et al. 2021), *Conidiobolus polytocus* (MZ436172, Nie et al. 2021), *Conidiobolus* sp. (MN640580, Sun et al. 2020), *Conidiobolus taihushanensis* (MZ436173, Nie et al. 2021), *Gigaspora rosea* (JQ693395, Nadimi et al. 2012), *Gigaspora margarita* (JQ041882, Pelin et al. 2012), *Harpochytrium* sp. JEL94 (AY182005, Bullerwell et al. 2003), *Harpochytrium* sp. JEL105 (AY182006, Bullerwell et al. 2003), *Microconidiobolus nodosus* (MW795365, Cai et al. 2021), *Mortierella verticillata* (AY863211, Seif et al. 2005), *Neoconidiobolus thromboides* (MW795364, Nie et al. 2021), *Rhizophagus fasciculatus* (KM586389, Nadimi et al. 2016), *Synchytrium endobioticum* (MK292696, van de Vossenberg et al. 2018), *Zancudomyces culisetae* (AY863213, Seif et al. 2005). *Colletotrichum acutatum* (KR349346, Kim et al. 2016), *Lactifluus hygrophoroides* (MH319477, Li et al. 2019), and *Yarrowia lipolytica* (CP017559, Magnan et al. 2016) were served as outgroups. The newly sequenced mitogenome is denoted in bold. Maximum-likelihood bootstrap values (1000 replicates) for each clade are indicated along the branches. The scale bar refers to 0.1 nucleotide substitutions per character.

The complete mitogenome sequence of *C. rhysosporum* (GenBank accession number MZ286627, Nie et al. 2019) is 46,756 base pairs in length, comprising two ribosomal RNA genes (*rnl* and *rns*), 27 tRNA genes, 15 conserved protein-coding genes (PCGs), and one free-standing ORF. The PCGs include ATP synthase (*atp6*, *atp8*, and *atp9*), NADH dehydrogenase (*nad1*, *nad2*, *nad3*, *nad4*, *nad4L*, *nad5*, and *nad6*), ubiquinol cytochrome c reductase (*cob*), cytochrome c oxidase (*cox1*, *cox2*, and *cox3*), and ribosomal protein (*rps3*). Twenty-seven introns were detected in seven genes, with one in *atp6* and *nad4*, two in *nad1*, three in *cox2*, five in *nad5*, seven in *cox1*, and eight in *cob* (Figure S2). The complete circular mitogenomes of *C. rhysosporum* are shown in Figure 2. In the phylogenetic tree (Figure 3), *C. rhysosporum* was located in the clade of Zoopagomycota, *C. rhysosporum* exhibited a close phylogenetic relationship with *C. heterosporum*, consistent with previous studies (Nie et al. 2019; Sun et al. 2020).

## Discussion and conclusions

In this article, we reported another complete mitogenome of the genus *Capillidium* with 46,756 bp in total length. The number and length of fungal mitogenomic introns have an implication for inferring the fungal mitogenome size (Friedrich et al. 2012; Torriani et al. 2014), and we noted that the number of introns (27) was fewer than that of *C. heterosporum* (30). The *rps3* gene encodes protein component of the ribosome for protein translation (Bullerwell et al. 2000), which was annotated in the mitogenome of *C. rhysosporum*. As a note, the mitogenome of *C. rhysosporum* is the second complete mitogenome in the family Capillidiaceae, which enhances our understanding of the evolution of this fungal group.

In summary, this study presents another complete mitochondrial genome within the family Capillidiaceae. We also conducted the phylogenetic analysis with other related entomophthoroid fungi based on the sequences of 14 protein coding genes, which reveal a close relationship with *C. heterosporum*. The comprehensive analysis of its mitochondrial genome contributes valuable insights into the phylogenetic relationships and evolutionary history of entomophthoroid fungi.

## Supplementary Material

Supplemental Material

## Data Availability

The data that support the findings of this study are openly available in GenBank of NCBI (https://www.ncbi.nlm.nih.gov). The accession number of mitogenome is MZ286627. The associated BioProject, SRA, and BioSample numbers are PRJNA1030606, SRR27393659, and SAMN37911642, respectively.

## References

[CIT0001] Al-Nakeeb K, Petersen TN, Sicheritz-Pontén T. 2017. Norgal: extraction and de novo assembly of mitochondrial DNA from whole-genome sequencing data. BMC Bioinformatics. 18(1):510–517. doi:10.1186/s12859-017-1927-y. 29162031 PMC5699183

[CIT0002] Andrews S. 2010. FastQC: a quality control tool for high throughput sequence data. Cambridge: Babraham Bioinformatics.

[CIT1001] Brefeld O. 1884. Conidiobolus utriculosus und minor. Untersuchungen aus der Gesammtgebiete der. Mykologie. 6(2):35–78.

[CIT0003] Bullerwell CE, Burger G, Lang BF. 2000. A novel motif for identifying *rps3* homologs in fungal mitochondrial genomes. Trends Biochem Sci. 25(8):363–365. doi:10.1016/s0968-0004(00)01612-1. 10916154

[CIT0004] Bullerwell CE, Leigh J, Forget L, Lang BF. 2003. A comparison of three fission yeast mitochondrial genomes. Nucleic Acids Res. 31(2):759–768. doi:10.1093/nar/gkg134. 12527786 PMC140500

[CIT0005] Cai Y, Nie Y, Wang ZM, Huang B. 2021. The complete mitochondrial genome of *Microconidiobolus nodosus* (*Entomophthorales*: *Ancylistaceae*). Mitochondrial DNA B Resour. 6(6):1743–1744. doi:10.1080/23802359.2021.1930219. 34104757 PMC8158263

[CIT0006] Caspermeyer J. 2016. MEGA evolutionary software re-engineered to handle today’s big data demands. Mol Biol Evol. 33(7):1887. doi:10.1093/molbev/msw074. 27189553

[CIT0007] Drechsler C. 1954. Two species of *Conidiobolus* with minutely ridged zygospores. Am J Bot. 41(7):567–575. doi:10.2307/2438717.

[CIT0008] Friedrich A, Jung PP, Hou J, Neuvéglise C, Schacherer J. 2012. Comparative mitochondrial genomics within and among yeast species of the *Lachancea* genus. PLOS One. 7(10):e47834. doi:10.1371/journal.pone.0047834. 23112855 PMC3480396

[CIT0009] Gryganskyi AP, Nie Y, Hajek AE, Hodge KT, Liu XY, Aadland K, Voigt K, Anishchenko IM, Kutovenko VB, Kava L, et al. 2022. The early terrestrial fungal lineage of *Conidiobolus*—transition from saprotroph to parasitic lifestyle. J Fungi. 8(8):789. doi:10.3390/jof8080789. PMC9409958 36012777

[CIT0010] Kim JO, Choi KY, Han JH, Choi IY, Lee YH, Kim KS. 2016. The complete mitochondrial genome sequence of the ascomycete plant pathogen *Colletotrichum acutatum*. Mitochondrial DNA A DNA Mapp Seq Anal. 27(6):4547–4548. doi:10.3109/19401736.2015.1101556. 26539901

[CIT0011] Li Q, Wang QF, Jin X, Chen ZQ, Xiong C, Li P, Liu QF, Huang WL. 2019. Characterization and comparative analysis of six complete mitochondrial genomes from ectomycorrhizal fungi of the *Lactarius* genus and phylogenetic analysis of the Agaricomycetes. Int J Biol Macromol. 121:249–260. doi:10.1016/j.ijbiomac.2018.10.029. 30308282

[CIT0012] Li Q, Wu P, Li LJ, Feng HY, Tu WY, Bao ZJ, Xiong C, Gui MY, Huang WL. 2021. The first eleven mitochondrial genomes from the ectomycorrhizal fungal genus (*Boletus*) reveal intron loss and gene rearrangement. Int J Biol Macromol. 172:560–572. doi:10.1016/j.ijbiomac.2021.01.087. 33476615

[CIT0013] Lowe TM, Eddy SR. 1997. tRNAscan-SE: a program for improved detection of transfer RNA genes in genomic sequence. Nucleic Acids Res. 25(5):955–964. doi:10.1093/nar/25.5.955. 9023104 PMC146525

[CIT0014] Magnan C, Yu J, Chang I, Jahn E, Kanomata Y, Wu J, Zeller M, Oakes M, Baldi P, Sandmeyer S. 2016. Sequence assembly of *Yarrowia lipolytica* strain W29/CLIB89 shows transposable element diversity. PLOS One. 11(9):e0162363. doi:10.1371/journal.pone.0162363. 27603307 PMC5014426

[CIT0015] Nadimi M, Beaudet D, Forget L, Hijri M, Lang BF. 2012. Group I intron-mediated trans-splicing in mitochondria of *Gigaspora rosea* and a robust phylogenetic affiliation of arbuscular mycorrhizal fungi with Mortierellales. Mol Biol Evol. 29(9):2199–2210. doi:10.1093/molbev/mss088. 22411852

[CIT0016] Nadimi M, Daubois L, Hijri M. 2016. Mitochondrial comparative genomics and phylogenetic signal assessment of mtDNA among arbuscular mycorrhizal fungi. Mol Phylogenet Evol. 98:74–83. doi:10.1016/j.ympev.2016.01.009. 26868331

[CIT0017] Nie Y, Wang L, Cai Y, Tao W, Zhang YJ, Huang B. 2019. Mitochondrial genome of the entomophthoroid fungus *Conidiobolus heterosporus* provides insights into evolution of basal fungi. Appl Microbiol Biotechnol. 103(3):1379–1391. doi:10.1007/s00253-018-9549-5. 30569217

[CIT0018] Nie Y, Yu DS, Wang CF, Liu XY, Huang B. 2020. A taxonomic revision of the genus *Conidiobolus* (*Ancylistaceae*, *Entomophthorales*): four clades including three new genera. Mycokeys. 66:55–81. doi:10.3897/mycokeys.66.46575. 32273794 PMC7136305

[CIT0019] Nie Y, Wang ZM, Zhao H, Liu XY, Huang B. 2021. Complete mitochondrial genome of *Neoconidiobolus thromboides* (*Entomophthorales*: *Ancylistaceae*). Mitochondrial DNA B Resour. 6(7):1840–1841. doi:10.1080/23802359.2021.1934167. 34124362 PMC8183557

[CIT0020] Nie Y, Zhao H, Wang ZM, Zhou ZY, Liu XY, Huang B. 2021. The gene rearrangement, loss, transfer and deep intronic variation in mitochondrial genomes of *Conidiobolus*. Front Microbiol. 12:765733. doi:10.3389/fmicb.2021.765733. 34858376 PMC8632527

[CIT0021] Nie Y, Zhao H, Wang ZM, Zhou ZY, Liu XY, Huang B. 2022. Two new species in *Capillidium* (*Ancylistaceae*, *Entomophthorales*) from China, with a proposal for a new combination. MycoKeys. 89:139–153. doi:10.3897/mycokeys.89.79537. 36760830 PMC9849098

[CIT0022] Paquin B, Lang BF. 1996. The mitochondrial DNA of *Allomyces macrogynus*: the complete genomic sequence from an ancestral fungus. J Mol Biol. 255(5):688–701. doi:10.1006/jmbi.1996.0056. 8636971

[CIT0023] Pelin A, Pombert JF, Salvioli A, Bonen L, Bonfante P, Corradi N. 2012. The mitochondrial genome of the arbuscular mycorrhizal fungus *Gigaspora margarita* reveals two unsuspected trans-splicing events of group I introns. New Phytol. 194(3):836–845. doi:10.1111/j.1469-8137.2012.04072.x. 22320438

[CIT0024] Posada D, Crandall KA. 1998. MODELTEST: testing the model of DNA substitution. Bioinformatics. 14(9):817–818. doi:10.1093/bioinformatics/14.9.817. 9918953

[CIT0025] Rambaut A. 2012. FigTree version 1.4.0. http://tree.bio.ed.ac.uk/software/figtree/.

[CIT0026] Ronquist F, Huelsenbeck JP. 2003. MrBayes 3: Bayesian phylogenetic inference under mixed models. Bioinformatics. 19(12):1572–1574. doi:10.1093/bioinformatics/btg180. 12912839

[CIT0027] Seif E, Leigh J, Liu Y, Roewer I, Forget L, Lang BF. 2005. Comparative mitochondrial genomics in zygomycetes: bacteria-like RNase P RNAs, mobile elements and a close source of the group I intron invasion in angiosperms. Nucleic Acids Res. 33(2):734–744. doi:10.1093/nar/gki199. 15689432 PMC548346

[CIT0028] Sun XR, Su D, Gui WJ, Luo F, Chen Y. 2020. Characterization and phylogenetic analysis of the complete mitochondrial genome of *Conidiobolus* sp. (*Entomophthorales*: *Ancylistaceae*). Mitochondrial DNA B Resour. 5(1):121–122. doi:10.1080/23802359.2019.1698340. PMC7721048 33366449

[CIT0029] Stamatakis A. 2014. RAxML version 8: a tool for phylogenetic analysis and post-analysis of large phylogenies. Bioinformatics. 30(9):1312–1313. doi:10.1093/bioinformatics/btu033. 24451623 PMC3998144

[CIT0030] Tambor JH, Ribichich KF, Gomes SL. 2008. The mitochondrial view of *Blastocladiella emersonii*. Gene. 424(1–2):33–39. doi:10.1016/j.gene.2008.07.031. 18721866

[CIT0031] Torriani SFF, Penselin D, Knogge W, Felder M, Taudien S, Platzer M, McDonald BA, Brunner PC. 2014. Comparative analysis of mitochondrial genomes from closely related *Rhynchosporium* species reveals extensive intron invasion. Fungal Genet Biol. 62:34–42. doi:10.1016/j.fgb.2013.11.001. 24240058

[CIT0032] Vaidya G, Lohman DJ, Meier R. 2011. SequenceMatrix: concatenation software for the fast assembly of multi-gene datasets with character set and codon information. Cladistics. 27(2):171–180. doi:10.1111/j.1096-0031.2010.00329.x. 34875773

[CIT0033] van de Vossenberg BTLH, Brankovics B, Nguyen HDT, van Gent-Pelzer MPE, Smith D, Dadej K, Przetakiewicz J, Kreuze JF, Boerma M, van Leeuwen GCM, et al. 2018. The linear mitochondrial genome of the quarantine chytrid *Synchytrium endobioticum*; insights into the evolution and recent history of an obligate biotrophic plant pathogen. BMC Evol Biol. 18(1):136. doi:10.1186/s12862-018-1246-6. 30200892 PMC6131824

[CIT0034] Watanabe M, Lee K, Goto K, Kumagai S, Sugita-Konishi Y, Hara-Kudo Y. 2010. Rapid and effective DNA extraction method with bead grinding for a large amount of fungal DNA. J Food Prot. 73(6):1077–1084. doi:10.4315/0362-028x-73.6.1077. 20537263

[CIT0035] Zhang YJ, Yang XQ, Zhang S, Humber RA, Xu J. 2017. Genomic analyses reveal low mitochondrial and high nuclear diversity in the cyclosporin-producing fungus *Tolypocladium inflatum*. Appl Microbiol Biotechnol. 101(23–24):8517–8531. doi:10.1007/s00253-017-8574-0. 29034434

[CIT0036] Zhang XY, Chen HM, Ni Y, Wu B, Li JL, Burzynski A, Liu C. 2023. Plant mitochondrial genome map (PMGmap): a software tool for comprehensive visualization of coding, non-coding and genome features of plant mitochondrial genomes. Authorea. doi:10.22541/au.169772240.03411454/v1. 38523350

