# Supplementary material for: The second complete mitochondrial genome of Capillidium rhysosporum within the family Capillidiaceae, Entomophthorales
Source: Mitochondrial DNA B Resour. 2024 Mar 11;9(3):332–7. doi: 10.1080/23802359.2024.2324938 (PMC10930110; doi:10.1080/23802359.2024.2324938)
Supplement: Supplemental Material [file TMDN_A_2324938_SM4759.pdf]

The Second Complete Mitochondrial Genome of *Capillidium rhysosporum* within the Family Capillidiaceae, Entomophthorales

Hanwen Lu[1], Yong Nie[2], Bo Huang[1*]

[1]Anhui Provincial Key Laboratory for Microbial Pest Control, Anhui Agricultural University, Hefei 230036, China

[2]School of Civil Engineering and Architecture, Anhui University of Technology, Ma'anshan 243002, China

Corresponding Authors:

B. Huang, e-mail: bhuang@ahau.edu.cn.

Annotation: the below two Supplementary Figure S1 and Figure S2 belong to this maunscript.

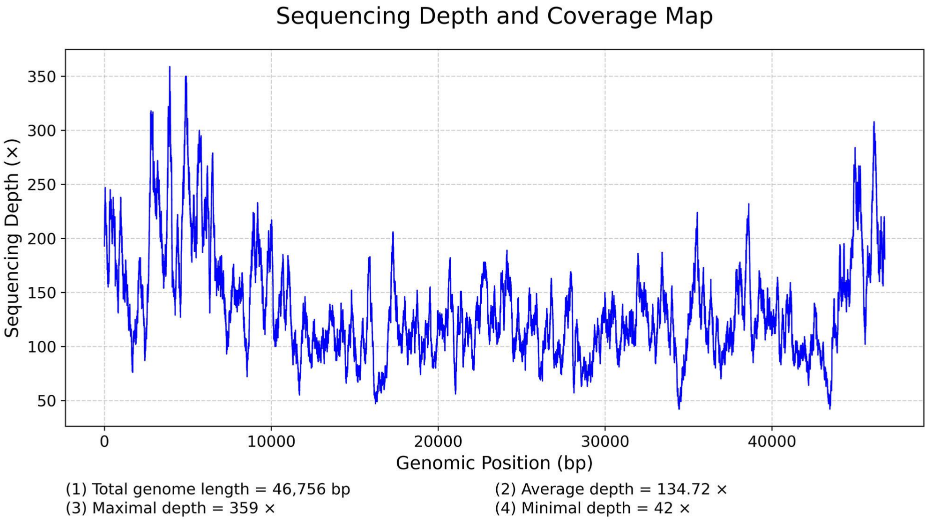

Supplementary Figure S1 Sequencing depth and coverage map of *Capillidium rhysosporum* mitochondrial genome.

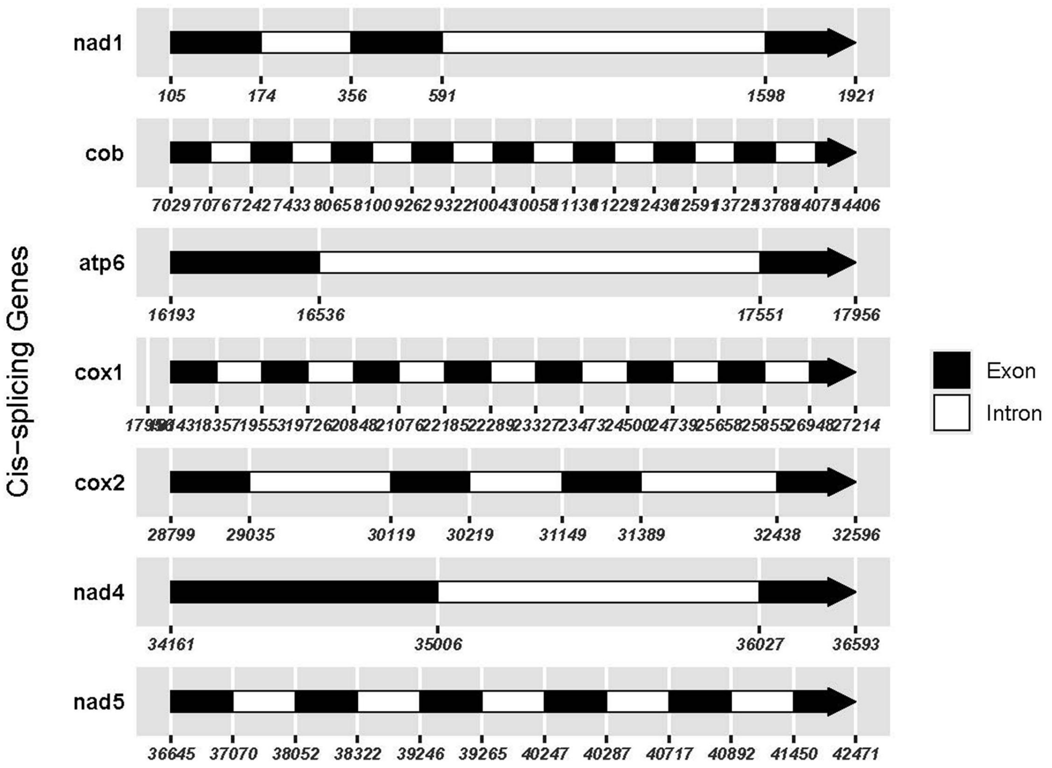

Supplementary Figure S2 Cis-splicing genes of the *Capillidium rhysosporum* mitochondrial genome.
